# The FOAM study: is Hysterosalpingo foam sonography (HyFoSy) a cost-effective alternative for hysterosalpingography (HSG) in assessing tubal patency in subfertile women? Study protocol for a randomized controlled trial

**DOI:** 10.1186/s12905-018-0556-6

**Published:** 2018-05-09

**Authors:** Joukje van Rijswijk, Nienke van Welie, Kim Dreyer, Machiel H. A. van Hooff, Jan Peter de Bruin, Harold R. Verhoeve, Femke Mol, Kimiko A. Kleiman-Broeze, Maaike A. F. Traas, Guido J. J. M. Muijsers, Arentje P. Manger, Judith Gianotten, Cornelia H. de Koning, Aafke M. H. Koning, Neriman Bayram, David P. van der Ham, Francisca P. J. M. Vrouenraets, Michaela Kalafusova, Bob I. G. van de Laar, Jeroen Kaijser, Miriam F. van Oostwaard, Wouter J. Meijer, Frank J. M. Broekmans, Olivier Valkenburg, Lucy F. van der Voet, Jeroen van Disseldorp, Marieke J. Lambers, Henrike E. Peters, Marit C. I. Lier, Cornelis B. Lambalk, Madelon van Wely, Patrick M. M. Bossuyt, Jaap Stoker, Fulco van der Veen, Ben W. J. Mol, Velja Mijatovic

**Affiliations:** 1 Department of Reproductive Medicine, VU University Medical Centre, Postal code, 7057 1007 MB Amsterdam, The Netherlands; 2 Department of Obstetrics and Gynaecology, Sint Franciscus Hospital, Kleiweg 500, 3045PM Rotterdam, The Netherlands; 3 Department of Obstetrics and Gynaecology, Jeroen Bosch Hospital, Henri Dunantstraat 1, 5223 GZ Den Bosch, The Netherlands; 4 Department of Obstetrics and Gynaecology, OLVG Oost, Oosterpark 9, 1091 AC Amsterdam, The Netherlands; 5 Centre for Reproductive Medicine, Department of Obstetrics and Gynaecology, Academic Medical Centre, Meibergdreef 9, 1105 AZ Amsterdam, the Netherlands; 6 Department of Obstetrics and Gynaecology, Flevo Hospital, Hospitaalweg 1, 1315 RA Almere, The Netherlands; 7 Department of Obstetrics and Gynaecology, Gelre Hospital, Albert Schweitzerlaan, 31 7334 DZ Apeldoorn, The Netherlands; 8 Department of Obstetrics and Gynaecology, Rivierenland Hospital, President Kennedylaan 1, 4002 WP Tiel, The Netherlands; 9 Department of Obstetrics and Gynaecology, Diakonessenhuis, Bosboomstraat 1, 3582 KE Utrecht, The Netherlands; 10 Department of Obstetrics and Gynaecology, Spaarne Gasthuis, Boerhaavelaan 22, 2035RC Haarlem, The Netherlands; 11 Department of Obstetrics and Gynaecology, Tergooi Hospital, Rijksstraatweg 1 1261 AN, Blaricum, The Netherlands; 12 Department of Obstetrics and Gynaecology, Amstelland Hospital, Laan van de Helende Meesters 8, 1186 AM Amstelveen, The Netherlands; 13 Department of Obstetrics and Gynaecology, Zaans Medical Centre, Kon, Julianaplein 58, 1502 DV Zaandam, Zaandam, The Netherlands; 14 Department of Obstetrics and Gynaecology, Martini Hospital Groningen, Van Swietenplein 1, 9700 Groningen, RB Netherlands; 15 Department of Obstetrics and Gynecology, Zuyderland Medical Center, Henri Dunantstraat 5, 6419 PC Heerlen, The Netherlands; 16 Department of Obstetrics and Gynaecology, Refaja Hospital, Boerhaavestraat 1, 9501 HE Stadskanaal, The Netherlands; 17 Department of Obstetrics and Gynaecology, OLVG West, Jan Tooropstraat, 164 1061AE Amsterdam, The Netherlands; 18 JDepartment of Obstetrics and Gynaecology, Ikazia medical center, Montessoriweg 1, 3083 AN Rotterdam, The Netherlands; 19 Department of Obstetrics and Gynaecology, IJsselland hospital, Prins Constantijnweg 2, 2906 ZC Capelle a/d Ijssel, The Netherlands; 20 Department of Obstetrics and Gynaecology, Gelre Hospital, Den Elterweg 77, 7207AE Zutphen, The Netherlands; 21 Department of Reproductive Medicine and Gynaecology, University Medical Centre Utrecht, Heidelberglaan 100, 3584 CX Utrecht, The Netherlands; 22 Department of Reproductive Medicine, Maastricht University Medical Centre, P. debeylaan, 25 6229HX Maastricht, The Netherlands; 23 Department of Obstetrics and Gynaecology, Deventer Hospital, Nico Bolkesteinlaan 75, 7416 SE Deventer, The Netherlands; 24 Department of Obstetrics and Gynaecology, Sint Antonius Hospital, Koekoekslaan 1, 3435 CM Nieuwegein, The Netherlands; 25 Department of Obstetrics and Gynaecology, Westfriesgasthuis, Maelsonstraat 3, 1624 NP Hoorn, The Netherlands; 26 Department of Epidemiology, Biostatistics and Bioinformatics, Academic Medical Centre, Meibergdreef 9, 1105 AZ Amsterdam, The Netherlands; 27 Department of Radiology and Nuclear Medicine, Academic Medical Centre, Meibergdreef 9, 1105 AZ Amsterdam, The Netherlands; 28 School of Paediatrics and Reproductive Health, The Robinson Research Institute and The South Australian Health and Medical Research Institute, Adelaide, Australia

**Keywords:** Hysterosalpingo foam sonography (HyFoSy), Hysterosalpingography (HSG), Fertility work-up, Tubal patency testing, Ongoing pregnancy, Cost-effectiveness, Subfertility, Randomized controlled trial, Budget impact

## Abstract

**Background:**

Tubal pathology is a causative factor in 20% of subfertile couples. Traditionally, tubal testing during fertility work-up is performed by hysterosalpingography (HSG). Hysterosalpingo-foam sonography (HyFoSy) is a new technique that is thought to have comparable accuracy as HSG, while it is less expensive and more patient friendly. HyFoSy would be an acceptable alternative for HSG, provided it has similar effectiveness in terms of patient outcomes.

**Methods/design:**

We aim to compare the effectiveness and costs of management guided by HyFoSy or by HSG. Consenting women will undergo tubal testing by both HyFoSy and HSG in a randomized order during fertility work-up. The study group will consist of 1163 subfertile women between 18 and 41 years old who are scheduled for tubal patency testing during their fertility work-up. Women with anovulatory cycles not responding to ovulation induction, endometriosis, severe male subfertility or a known contrast (iodine) allergy will be excluded. We anticipate that 7 % (*N* = 82) of the participants will have discordant test results for HyFoSy and HSG. These participants will be randomly allocated to either a management strategy based on HyFoSy or a management strategy based on HSG, resulting in either a diagnostic laparoscopy with chromopertubation or a strategy that assumes tubal patency (intrauterine insemination or expectant management). The primary outcome is ongoing pregnancy leading to live birth within 12 months after randomization. Secondary outcomes are patient pain scores, time to pregnancy, clinical pregnancy, miscarriage rate, multiple pregnancy rate, preterm birth rate and number of additional treatments. Costs will be estimated by counting resource use and calculating unit prices.

**Discussion:**

This trial will compare the effectiveness and costs of HyFoSy versus HSG in assessing tubal patency in subfertile women.

**Trial registration:**

Dutch Trial Register (NTR 4746, http://www.trialregister.nl). Date of registration: 19 August 2014.

## Background

Subfertility, defined as the inability to conceive within 12 months of unprotected intercourse, affects 1 out of 6 couples trying to get pregnant [1]. Traditionally, the diagnostic work-up for subfertility includes tests to assess tubal status, among which hysterosalpingography (HSG) and diagnostic laparoscopy with chromopertubation (DLS) are the most established tests [2]. HSG is still the test of first choice during the fertility work-up in many clinics in the Netherlands. In case bilateral tubal pathology is suspected, a DLS is performed which is considered the clinical reference standard. DLS is an invasive test under general anesthesia that allows direct visualization of the pelvis, including fallopian tubes, ovaries and uterus. However, there is a risk for visceral damage, intra-abdominal bleeding and risks related to general anesthesia. Initially, hysterosalpingo-contrast sonography (HyCoSy) has been proposed as an alternative for HSG as a first line office tubaI patency test. The accuracy of HyCoSy is comparable to that of HSG [3, 4]. However, the commonly used echogenic medium for HyCoSy Microcrystalline suspension (Echovist®, Schering AG, Berlin, Germany), is no longer available. In 2011, hysterosalpingo-foam sonography (HyFoSy) was introduced as a new technique for tubal patency testing and an alternative for HyCoSy [5]. This imaging technique is comparable to HyCoSy, but it uses foam instead of gel.

The advantages of HyFoSy over HSG are manifold; with HyFoSy there is no radiation exposure which makes it a more patient friendly examination compared to HSG. In addition, the HyFoSy procedure is less painful as well as less time consuming compared with HSG [6] and a HyFoSy can be performed by the gynecologist during regular office hours, establishing the fertility work-up in a one stop clinic.

Two small observational cohort studies have been published reporting on the diagnostic performance of HyFoSy [7, 8]. An observational cohort study in 20 subfertile women found a 100% agreement between tubal patency data according to HyFoSy testing and DLS [8]. In a prospective observational cohort study in 73 women, HyFoSy was able to demonstrate two-sided tubal patency in 57 women, but technically unsuccessful in terms of inadequate filling of the uterine cavity in 6 women, while two-sided tubaI patency could not be demonstrated in 10 other women. A subsequent HSG in those 10 participants confirmed tubal occlusion in 5 (7%). In the remaining 5 women (7%) there was discordance between HyFoSy and HSG. The authors concluded that using HyFoSy as first line tubal test during the fertility work-up could avoid an HSG in about 78% of the cases (57 out of 73) [7]. There are no large studies that assess HyFoSy and HSG.

HyFoSy would be an acceptable alternative to HSG for tubal patency testing during fertility work-up in subfertile women if it leads at least to similar outcomes, in terms of live births, but lower costs. This randomized trial with a discordancy design compares two management strategies, one in which management is guided by HyFoSy and one that is guided by HSG.

## Methods / design

### Design

**Fig. 1:**
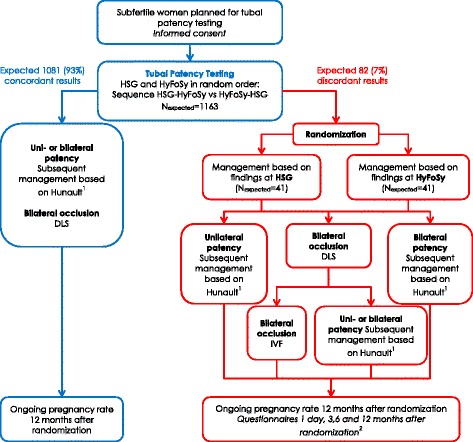
Flowchart FOAM study. ^1^Management based on the prognostic model of Hunault for natural conception: > 30%: 6 months expectant, followed by 6 cycles intrauterine insemination (IUI); < 30%: 6 cycles IUI, followed by in-vitro fertilization (IVF). ^2^Questionnaires: Short-Form-36 (SF-36), Health and Labour Questionnaire (HLQ)

The FOAM study is a multicenter prospective comparative study with a randomized controlled trial design (Fig. 1) [9]. It will be performed in hospitals that collaborate within the Dutch Consortium for Studies in Women’s Health and Reproduction. Participating centers can be district, teaching or university hospitals. Gynecologists and/or ultrasound technicians will be trained in their center in the performance of the HyFoSy by one of the physicians familiar with HyFoSy (VM, KD or JvR).

### Participants / eligibility criteria

Subfertile women, between 18 and 41 years old, who are scheduled for tubal patency testing as part of the fertility work-up are eligible for inclusion. Women with anovulatory cycles not responding on ovulation induction, endometriosis, severe male factor (Total motile sperm count < 1 × 10^6^/ml) or a known contrast (iodine) allergy will be excluded.

### Study procedures

All consenting participants will be scheduled for both HyFoSy and HSG. The order of these tests will be determined by randomization. Both tubal patency tests will be done within the two weeks of the follicular phase of the cycle after complete cessation of menstrual bleeding. The physician performing the HSG will be blinded for the results of the previously performed HyFoSy and vice versa. Test results of HyFoSy and HSG will be classified as normal, one-sided tubal pathology or double-sided tubal pathology.

lf the results of both tubal patency tests are concordant, the planned fertility treatment will be based on the test results in accordance with the current Dutch guideline [2]. Participants with one or two patent tubes at HyFoSy and HSG will be treated according to their prognosis for natural conception based on the model of Hunault [10]. In case the chance of natural conception within the following 12 months exceeds 30%, participants will be counseled for expectant management for 6-12 months. In case the chance is less than 30%, participants will be treated with intrauterine insemination (IUI) eventually followed by in vitro fertilization (IVF). In case HyFoSy and HSG are concordant in the diagnosis of suspected bilateral tubal occlusion, participants will be scheduled for DLS, followed by IVF in case bilateral tubaI occlusion is confirmed. In case of one-sided or two-sided patency during DLS the subsequent fertility treatment will also be based on the Hunault prognosis for natural conception.

Participants in whom the results of the tubaI patency tests HyFoSy and HSG are discordant will subsequently be included in a randomized trial in which they will be randomized between management based on the results of HyFoSy or management based on the results of HSG.

Consequently, in case unilateral or bilateral patent tubes are observed by the allocated tubal patency test, subsequent management will be according to the assumption of tubal patency. This will be 6 to 12 months expectant management in case of a probability of more than 30% for a natural conception according to the model of Hunault [10], while in case of a probability of less than 30% for spontaneous conception, IUI will be recommended. If bilateral occlusion is observed by the allocated tubal patency test, subsequent management will be a DLS, and if tubal occlusion is confirmed, the next step will be IVF. In case DLS shows tubal patency of at least one tube, subsequent management will be just as in the trial arm assuming tubal patency, i.e. expectant management or IUI.

#### HSG procedure

During an HSG approximately 10 cm^3^ of water soluble or oil soluble iodinated contrast medium (depending on local protocol of the participating hospitals) will be infused in the uterine cavity and fallopian tubes through the use of a Semm cup (a plastic vacuum cup that will be placed on the cervix) or balloon catheter. The contrast medium will be visible on X-ray. During instillation of the contrast medium into the uterine cavity and fallopian tubes a series radiographs (6-8) will be made to establish the patency of the fallopian tubes.

#### HyFoSy procedure

During a HyFoSy procedure approximately 10 cm^3^ of foam will be introduced, through a little cervical balloon-less applicator, into the uterine cavity. This applicator is connected to a syringe with foam. This foam is created by rigorously mixing 5 ml ExEm-gel® (containing hydroxyethyl cellulose and glycerol, IQ Medical Ventures BV, Delft, The Netherlands) with 5 ml of purified water in a 10 ml syringe. This recipe has turned out to be excellent for creating foam that is sufficiently stable to show echogenicity for at least 5 min and for providing sufficient fluid to pass through patent tubes. [5] During infusion of the foam into the uterine cavity a transvaginal ultrasound will be made, to show whether the fallopian tubes are patent.

#### DLS procedure

Diagnostic laparoscopy will be performed under general anesthesia in a daycare setting. The technique used is a double-puncture technique. The optic will be introduced through a trocar in the belly button. A suprapubic trocar is placed to make manipulation possible. A Foley catheter is placed in the uterine cavity. During the laparoscopy methylene blue is introduced into the uterine cavity through the Foley catheter and the patency of the fallopian tubes can be confirmed under direct vision by the optic. The amount of methylene blue injected will be variable, depending on the time necessary to assess tubal function.

##### Recruitment, consent and randomization, collection of data

Eligible women receive oral and written information during their regular outpatient visit by the attending gynecologist or fertility doctor. Women will be contacted by telephone for further information by the investigator. Women who agree to participate will be asked to sign written informed consent, of which they will receive a copy at their next visit, when the informed consent form will also be signed by the investigator, supervising gynecologist, the attending registrar or fertility doctor. Women who decline randomization will be offered the standard tubal patency testing with HSG or DLS, depending on the standard management policy in the clinic. Women refusing participation are registered.

Consenting eligible women undergo a HyFoSy as well as an HSG in a random order. Randomization is stratified for each center and will be performed after baseline data have been entered in a central web-based system that is available in our research consortium (ALEA) with the use of a permuted block design. Randomization is performed with only initials and year of birth of the participants. Linking personal data to the study number can only be performed in the local participating centers. Written informed consent forms are stored in every center in a lockable room. All forms and data will be archived for 15 years in the participating centers.

When there is discordance between the results of HSG and HyFoSy participants will be randomly allocated by the web based randomization program (ALEA) for management based on either HyFoSy or HSG.

After inclusion, all measurements will be systematically recorded using an electronic Clinical Report Form. These electronic forms will be stored in a digital database. Data are handled confidentially and, whenever possible anonymously. A subject identification code list will be used to link the data to the subject, where it is necessary to be able to trace data to an individual subject. The code will not be based on the woman’s initials and birth date, the key to the code will be safeguarded by the local investigator. The handling of personal data will comply with the Dutch Personal Data Protection Act (in Dutch: De Wet Bescherming Persoonsgegevens, Wbp).

All participants will receive a questionnaire about pregnancy outcomes 12 months after randomization. Participants with discordant test results will receive digital online secured questionnaires on day 1 after randomization and after, 3, 6 and 12 months respectively. Questionnaires include information on quality of life (SF-36: Short-Form-36) [11, 12] and absence from work (HLQ: Health and Labour Questionnaire) [13].

All adverse events reported spontaneously by the subject or observed by the investigator or his staff will be recorded. All Serious Adverse Events (SAE’s) will be reported to the Medical Ethics Committee.

The recruitment of participants started in June 2015, it is expected to conclude August 2018.

##### Outcome measures

The primary outcome for the comparison of the two strategies is ongoing pregnancy leading to live birth within 12 months after inclusion. Ongoing pregnancy rate is defined as an intrauterine pregnancy with a positive heartbeat during ultrasound examination between 10 and 12 weeks of pregnancy. Secondary outcomes are pain scores (measured by Visual Analogue Scale (VAS) scores), time to pregnancy, clinical pregnancy (defined as an ultrasound visible gestational sac with or without heartbeat), miscarriage (defined as the presence of non-vitality on ultrasound or spontaneous loss off pregnancy), multiple pregnancy (defined as a pregnancy of two or more fetus), preterm birth rate (defined as a delivery before 37 weeks of pregnancy), quality of life and absence of work.

#### Statistical analysis

We will estimate and compare the proportion of women with an ongoing pregnancy leading to live birth within 12 months after inclusion for two strategies: one in which management in subfertile women undergoing tubal patency testing is guided by the results of HSG and a second strategy, in which management is guided by the results of HyFoSy. To estimate this proportion, we will have to combine the outcomes observed in the concordant group, in which management will be identical for the two strategies, and in the discordant group, in which women are randomly allocated to management based on HSG or management based on HyFoSy.

The proportion of women with an ongoing pregnancy leading to live birth for each strategy will be a weighted average of the proportions observed in two groups: the concordant group and the discordant group, where the patients in the discordant group have to be counted twice, to account for the randomization, since in half of the women in the discordant group management will be guided by the other test. Lu and Gatsonis [14] have shown that this estimate is unbiased, and have provided a closed form for the variance of this estimate. They have also shown that this paired design is more efficient than an unpaired comparison of test & management strategies, especially if the proportion of women with discordant results is low. The effectiveness of management based on HyFoSy relative to management based on HSG will be expressed as a relative risk and as an absolute difference, each with 95% confidence intervals. SPSS will be used to perform the statistical analysis.

The primary analysis will be a non-inferiority test, in which we want to exclude a decrease of 2% when relying on the HyFoSy results instead of on the HSG. No interim analysis will be performed.

#### Sample size

We assume a 50% ongoing pregnancy rate within 12 months after tubal testing, with no difference between HSG or HyFoSy. When using a non-inferiority test at a 5% significance level, the total sample size will be guided by the fraction *f* of participants with discordant results (these will be randomized). In the randomized subgroup, the corresponding non-inferiority margin will then correspond to 2% divide by the fraction with discordant results *f* [14]. Assuming the fraction with discordant results is 7% [7], the non-inferiority margin in the discordant results will be 29%. To achieve 80% power to reject inferiority, we would then need to randomize 74 women with discordant results, and the total number to be included will be 1057 (74 divide by 7%). To account for a 10% loss to follow-up, we will include 1163 participants, resulting in 82 participants with discordant results.

##### Economic evaluation

The economic analysis will be performed alongside the clinical trial and will estimate costs from a third-party payer as well as from a societal perspective. A distinction will be made between costs of medical interventions (direct costs) and costs resulting from productivity losses (indirect costs), obtained from the HLQ questionnaires. Standardized units will be calculated for all centers based on actual resource use made during the trial. The economic evaluation will be designed as a cost-effectiveness analysis with the costs per ongoing pregnancy within 12 months as the primary outcome. The cost-effectiveness of each strategy will be presented as cost per live birth. A discounting rate of 4% will be used in the analysis. The incremental cost-effectiveness ratio (ICER) of a strategy based on HyFoSy as compared to a strategy based on HSG will be estimated as the ratio between difference in costs between the strategies and the difference in pregnancy rates, and reflects the extra costs required to obtain one additional live birth. The economic analyses will be presented in a separate report.

##### Ethical consideration

This study is approved by the National Central Committee on Research involving Human Subjects (CCMO – NL50484.029.14), by the ethics committee of the VU Medical Centre Amsterdam (Ref. No. 2014/454) and by the boards of all participating hospitals. The trial is registered in the Dutch Trial Register (NTR 4746, http://www.trialregister.nl).

## Discussion

HSG is the most widely used outpatient tubal test during fertility work-up. It was introduced in 1914 and still serves as an accurate diagnostic test, but may be painful, implies exposure to ionizing radiation and is expensive. HyFoSy is a new technique and proposed as a more patient friendly alternative for HSG as a first line office tubal patency test. No large trials have been published comparing HSG with HyFoSy. lf HyFoSy is as accurate as HSG in diagnosing tubal patency, it will lead to comparable management decisions and similar pregnancy outcomes and HSG could be substituted by HyFoSy for tubal patency testing during fertility work-up. Since approximately 20,000 HSGs are performed each year in the Netherlands and based on a cost difference between HyFoSy and HSG of around €100, replacing HSG by HyFoSy could result in substantial cost reduction.

The use of two physicians for the two tubal patency tests can be a logistical challenge for smaller district centers. However, this blinding is an essential aspect for the objectivity of test results of the whole study population and the strategy comparison of the randomization in case of discordance. Further randomization and allocation concealment through a web based randomization program reduces the chance for selection bias.

The final choice between HyFoSy and HSG will also depend on the direct therapeutic effect of both procedures. A recent large randomized clinical trial confirmed the long-stated hypothesis that HSG with oil-soluble contrast directly improves ongoing pregnancy and live birth rates [15]. Thus, if the FOAM study shows that HyFoSy is cost-effective over HSG in terms of diagnostic accuracy, the next question to be answered is if tubal flushing with oil after HyFoSy improves pregnancy rates.

The additional burden for women included in the study follows from undergoing one additional tubal patency test. This can lead to reluctance in taking part in the FOAM study. Offering the two tests on the same day, explaining women that HyFoSy is a more patient-friendly and less painful examination then HSG [6] and pointing out the potential benefit of HyFoSy for patients in the future, might overcome this.

If this trial shows that the fertility work-up with tubal testing based on HyFoSy is an efficient and effective alternative to HSG, the results may lead to evidence-based changes in national and international guidelines.

## Abbrevations

DLS: Diagnostic laparoscopic with chromopertubation
HLQ: Health and Labour Questionnaire
HSG: Hysterosalpingography
HyCoSy: Hysterosalpingo-contrast sonography
HyFoSy: Hysterosalpingo Foam Sonography
ICER: Incremental cost-effectiveness ratio
IUI: Intra- Uterine Insemination
IVF: In vitro fertilization
RCT: Randomized controlled trial
SF-36: Short-Form-36
VAS: Visual Analogue Scale
